# Correlation of Multi-Detector Computed Tomography and Intraoperative Variations of the Celiac Trunk and Hepatic Artery in Resectable Hepatobiliary Pancreatic Cancers

**DOI:** 10.7759/cureus.12106

**Published:** 2020-12-16

**Authors:** Satish Subbiah Nagaraj, Lileswar Kaman, Divya Dahiya, Krishna Ramavath, Naveen Kalra, Arunanshu Behera

**Affiliations:** 1 Department of General Surgery, Postgraduate Institute of Medical Education and Research, Chandigarh, IND; 2 Department of Radiology, Postgraduate Institute of Medical Education and Research, Chandigarh, IND

**Keywords:** celiac artery variations, mdct angiography, hepatobiliary pancreatic malignancies, surgery

## Abstract

Introduction

Knowledge of celiac artery variations is imperative to perform complex hepato-biliary pancreatic surgical procedures to avoid inadvertent complications. Multi-detector computed tomographic (MDCT) angiography aids in detecting these variations preoperatively. Surgical confirmation is considered the gold standard.

Aims and objectives

Preoperative assessment of celiac artery variations by MDCT angiography and surgical confirmation intraoperatively in resectable hepato-biliary pancreatic cancers.

Patients and methods

MDCT angiography was performed in 40 patients with clinical evidence of resectable hepato-biliary-pancreatic cancers. Three dimensional (3D) reconstructions were performed to confirm the celiac artery variations. Surgery was performed as per the institute’s protocol in all these patients for resection of tumor and confirmation of celiac artery anatomy. Variations were confirmed surgically that were identified through imaging.

Results

MDCT angiography identified normal trifurcated celiac artery anatomy in 33 (82.5%) patients and variant anatomy in seven (17.5%) patients. The most common variation was a replaced right hepatic artery (r-RHA) from the superior mesenteric artery (SMA) in four (10%) of patients. A replaced left hepatic artery (r-LHA) from the celiac trunk, a common hepatic artery (CHA) from the abdominal aorta, and an accessory right hepatic artery (ac-RHA) from the proper hepatic artery itself were identified in one (2.5%) patient each, respectively. All these findings were confirmed intraoperatively. There was a 100% statistical correlation between imaging and surgical findings.

Conclusion

Surgical confirmation of radiological data of celiac artery variations is the gold standard to avoid disastrous complications such as inadvertent vascular bleeds, biliary injuries, and hepatic necrosis. Since the presence of variations warrants the preservation or excision of the arterial system without oncological compromise and minimizing surgical complications.

## Introduction

Appreciation and knowledge of celiac trunk and hepatic artery anatomy and its variations is imperative to perform complex hepato-biliary pancreatic (HBP) surgical procedures, which involves dissection around these vascular structures. This knowledge aids to avoid disastrous complications such as inadvertent vascular bleeds, biliary injuries, and ischemic hepatic failure. Anatomical variations of the celiac artery are reported in the range of 10%-49%, which occur during embryological differentiation in fetal life [1-5]. These variations were historically identified by cadaveric dissections, which lead to the proposal of multiple classification systems [2,5]. Among these, Michel’s classification is the most commonly accepted [2].

With the advent of technological advances in radiology, the most novel and non-invasive approach in recent times is multi-detector computed tomography (MDCT) angiography [4,6-8]. The absence of surgical confirmation of radiological data limits its beneficiary to patients directly [9]. Surgical identification is now the proven gold standard to detect these variations [4,10-13]. Hence, this study was aimed to perform a non-invasive preoperative assessment of celiac artery variations by MDCT angiography and its intraoperative confirmation surgically. Since the presence of variations warrants the preservation or excision of the arterial system without oncological compromise and minimizing surgical complications.

## Materials and methods

This was a prospective observational study done over a period of 18 months (July 2015-November 2016). All patients with clinical evidence of HBP malignancies were evaluated by MDCT abdomen (128 and 256 slices) with its due contrast injection protocol of our institute. Only resectable malignancies based on the American Joint Committee on Cancer (AJCC) guidelines [14] were included for further evaluation on angiographic multi-planar reconstructions (MPR), as well as three-dimensional (3D) reconstructions. The images were analyzed and processed by experienced radiologists to notify normal and variant celiac trunk and hepatic artery anatomy based on Michel’s classification [2]. After proper informed consent, patients were preoperatively optimized to undergo surgical resection and lymphadenectomy with preoperative imaging in mind. Dissection was proceeded as per the institute’s surgical protocol to delineate the anatomy of the celiac trunk and hepatic artery without oncological compromise. Variant anatomy was specially delineated and notified. Correlation of MDCT angiography and intraoperative anatomy was done.

## Results

Seventy-five patients with clinical evidence of HBP malignancies were evaluated by MDCT abdomen, out of which 35 patients were excluded in view of unresectability based on AJCC-8 staging [14]. The remaining 40 patients were further evaluated initially by MDCT angiographic reconstructions and intraoperative findings were confirmed surgically. Demographic and HPB cancer data are depicted in Table 1.

**Table 1:** Demography and HBP cancer data HBP: hepato-biliary pancreatic

Number of Patients	40
Age Range	28-75 years
Mean Age+ Standard Deviation	54.30 + 10.86 years
Male	19 (47.5%)
Female	21 (52.5%)
Carcinoma Gall Bladder	9 (22.5%)
Carcinoma Head of Pancreas	8 (20%)
Periampullary Carcinoma	7 (17.5%)
Distal Cholangiocarcinoma	6 (15%)
Hilar Cholangiocarcinoma	4 (10%)
Hepatocellular Carcinoma	2 (5%)
Carcinoma Uncinate Process of the Pancreas	1 (2.5%)
Duodenal Gastro-Intestinal Stromal Tumour	1 (2.5%)
Neuro Endocrine Tumour Pancreas with Left Lobe Liver Metastasis	1 (2.5%)
Solid Papillary Neoplasm of the Pancreas	1 (2.5%)

**Figure 1:**
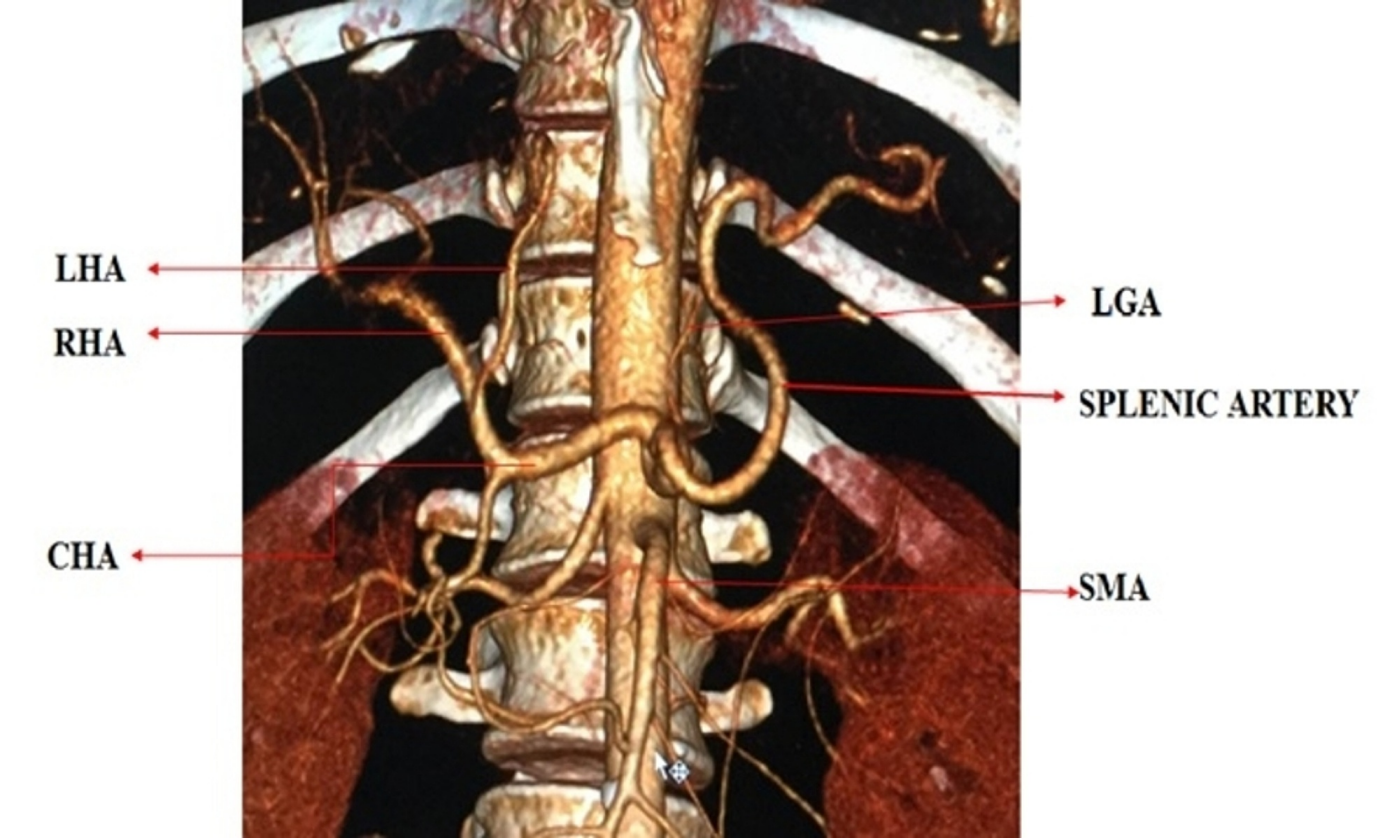
3D reconstruction of normal trifurcation of the celiac artery LHA: left hepatic artery; LGA: left gastric artery; RHA: right hepatic artery; CHA: common hepatic artery; SMA: superior mesenteric artery

**Figure 2:**
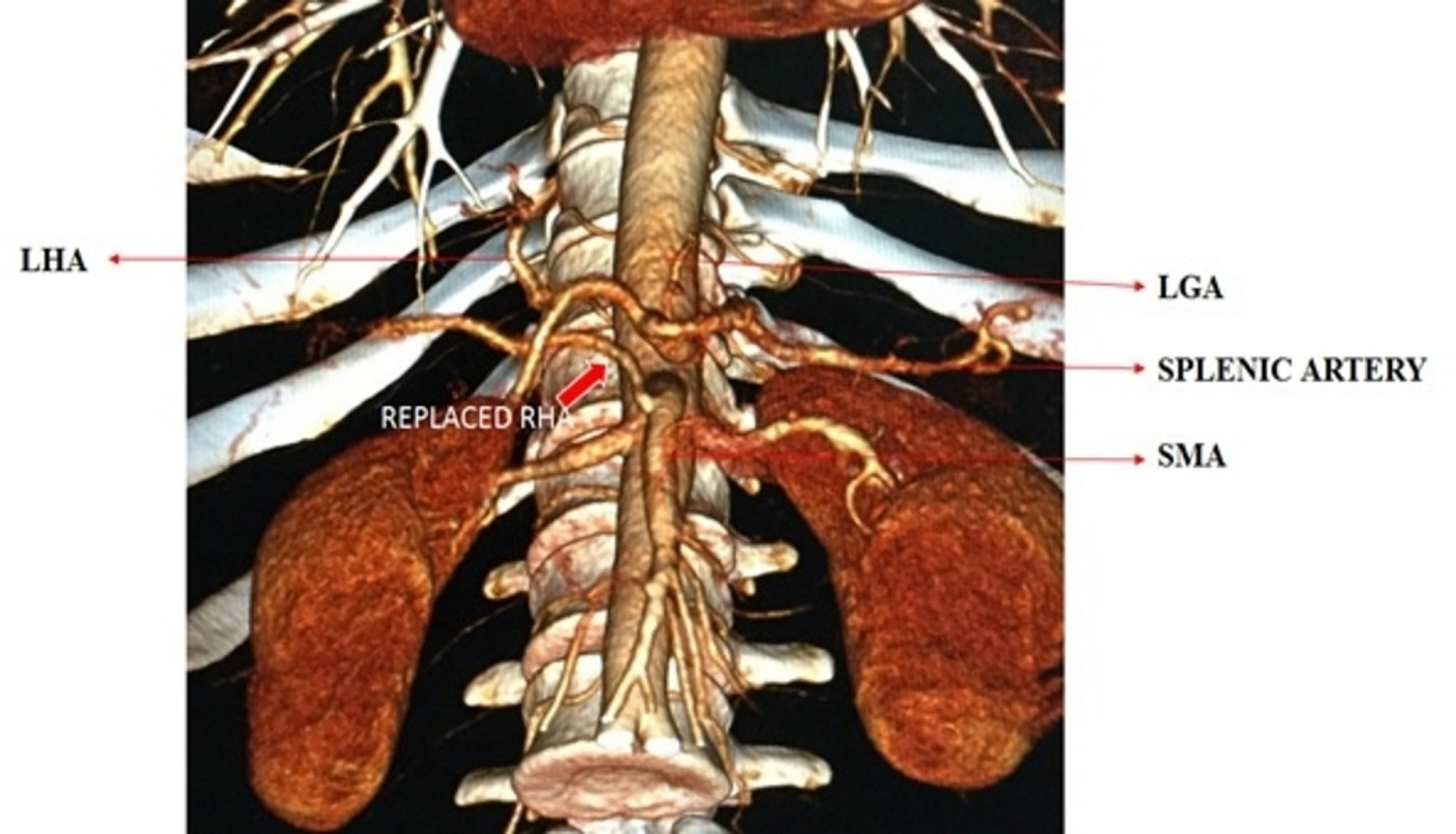
3D reconstructed image of the replaced right hepatic artery from the superior mesenteric artery r-RHA: replaced right hepatic artery (bold red arrow); SMA: superior mesenteric artery; LHA: left hepatic artery; LGA: left gastric artery

**Figure 3:**
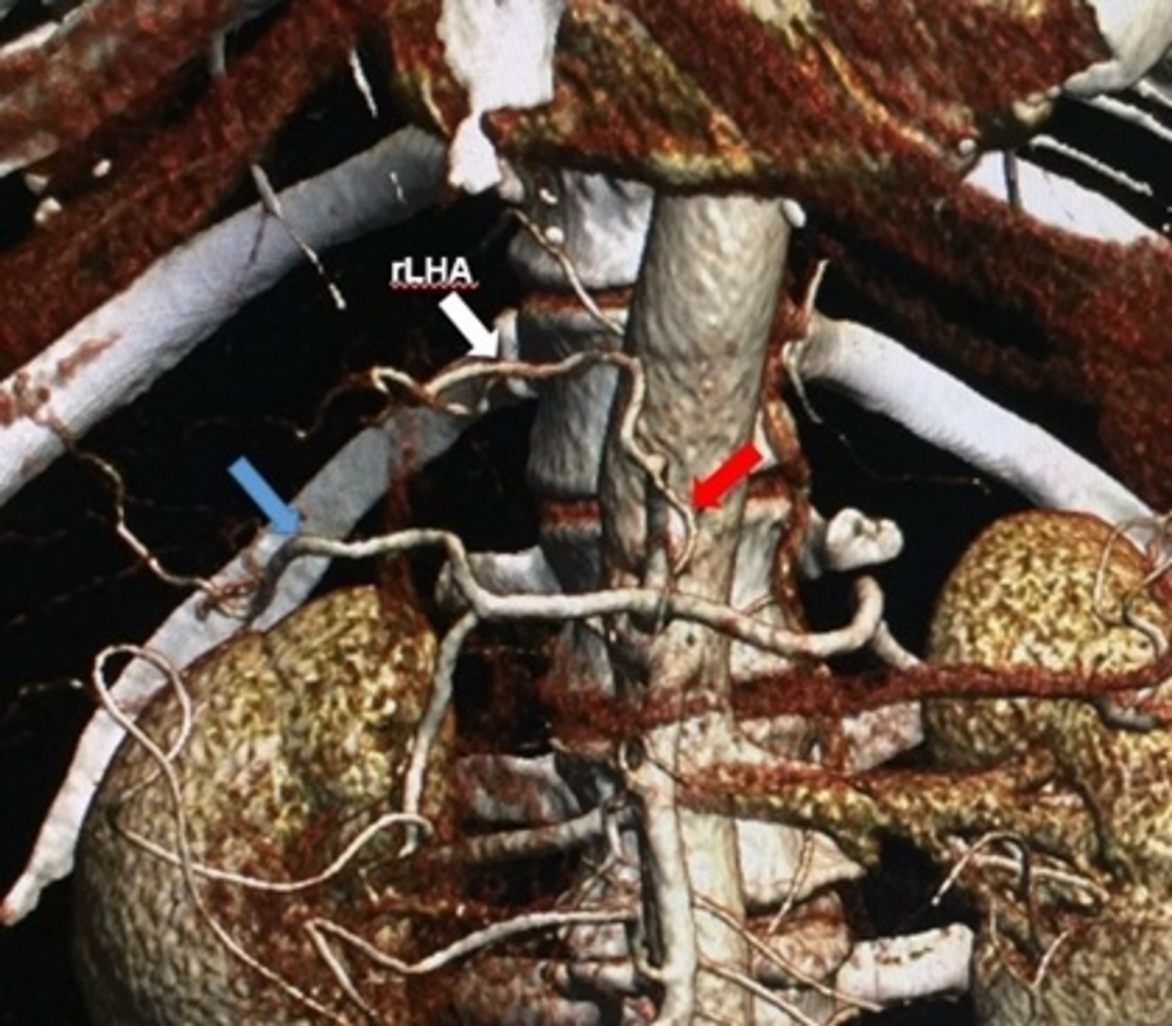
3D reconstructed image showing replaced left hepatic artery from the celiac trunk r-LHA: replaced left hepatic artery (white arrow); RHA: right hepatic artery (blue arrow); LGA: left gastric artery (red arrow)

**Figure 4:**
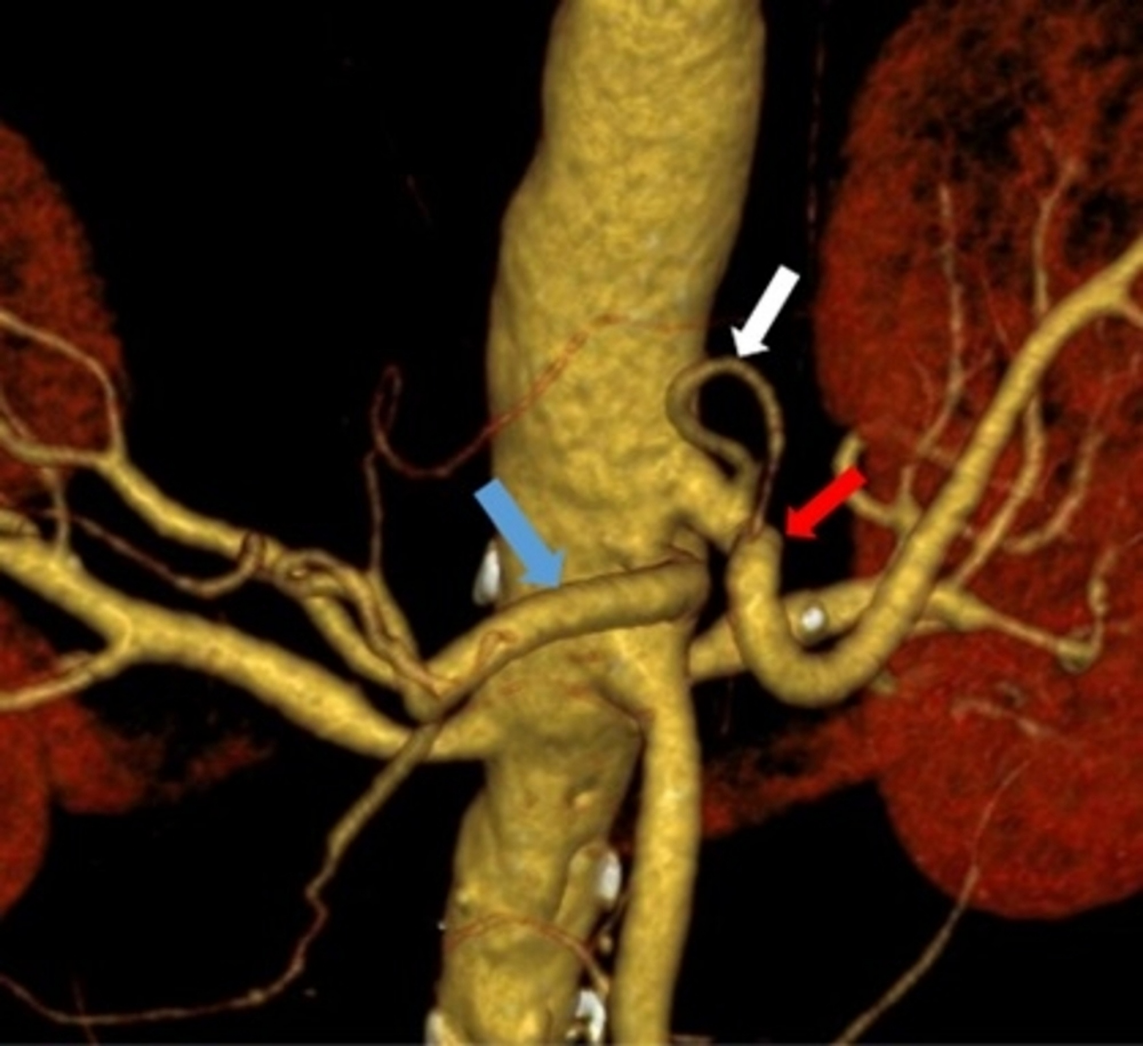
3D reconstructed image of common hepatic artery arising from the abdominal aorta, left gastric artery, and splenic artery from the abdominal aorta (gastrosplenic trunk) CHA: common hepatic artery (blue arrow); LGA: left gastric artery (white arrow); splenic artery (red arrow)

**Figure 5:**
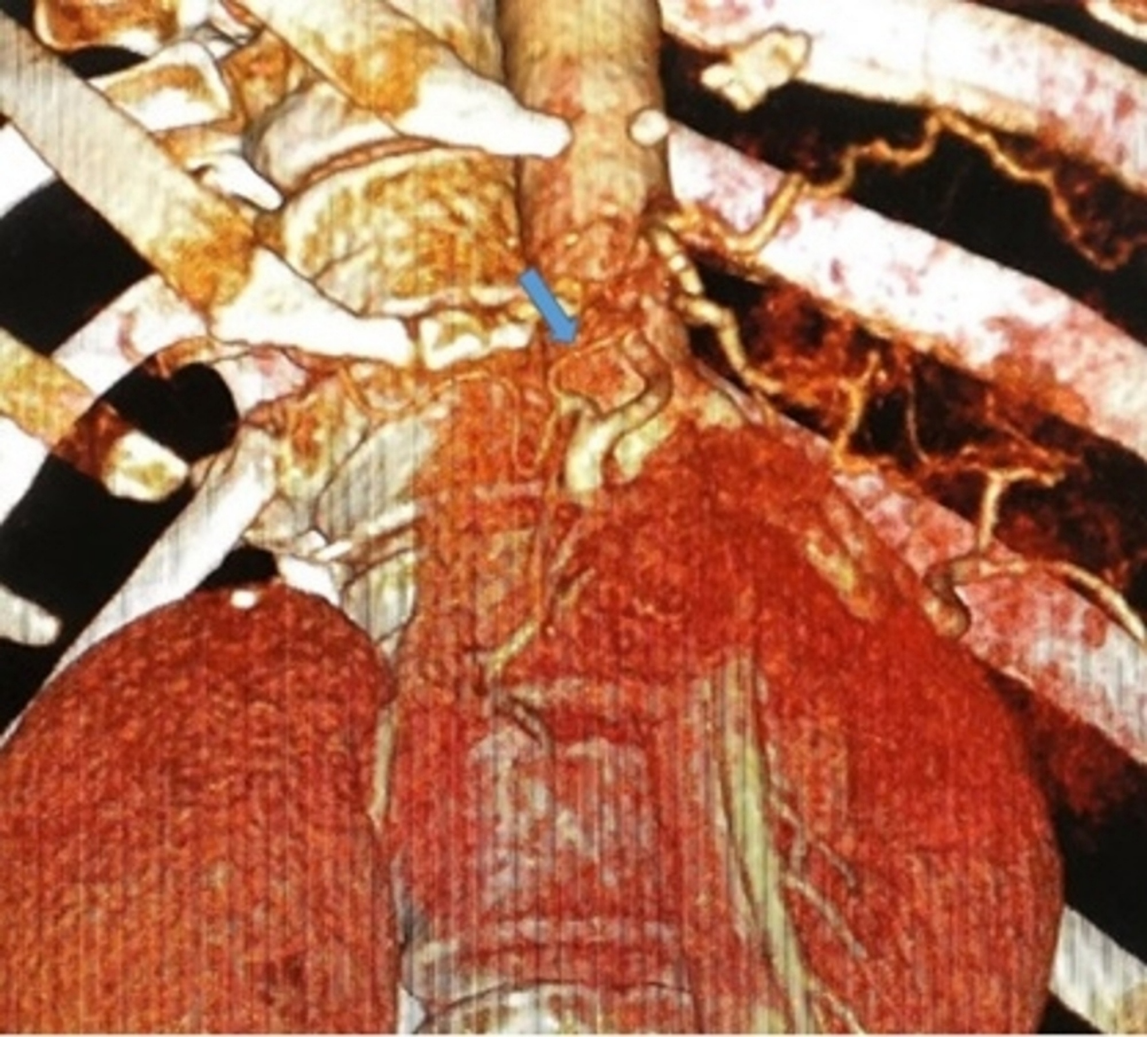
3D reconstructed image showing the accessory right hepatic artery from the proper hepatic artery ac-RHA: accessory right hepatic artery (blue arrow)

MDCT angiography identified the normal trifurcated anatomy of the celiac artery in 33 (82.5%) patients (Figure 1). Variant anatomy was identified in seven (17.5%) patients (Table 2). Most of the variations were found in males (6 out of 7 variations). The most common anatomical variation was a replaced right hepatic artery (r-RHA) from the superior mesenteric artery (SMA) in four (10%) patients (Figure 2). The replaced left hepatic artery (r-LHA) originating directly from the celiac trunk was found in one (2.5%) patient (Figure 3). In one patient (2.5%), the common hepatic artery (CHA) originated directly from the abdominal aorta with the gastro-splenic trunk as a separate root (Figure 4). An accessory right hepatic artery (ac-RHA) arising from the proper hepatic artery itself was found in one (2.5%) patient (Figure 5). There was no evidence of celiac artery stenosis, obstruction, and aneurysm in any of these patients. All 40 patients were subjected to surgery for resection of the tumor and lymphadenectomy. The intraoperative findings of all patients were recorded and were found exactly similar to MDCT angiography findings. There was no vascular injury or oncological compromise surgically in patients identified with variations due to a clear preoperative vascular roadmap and tumor facial planes. Difficulty in tracing the origin of a variant artery identified intraoperatively was avoided due to preoperative pathway confirmation in MDCT angiography.

**Table 2:** Prevalence and correlation of MDCT and surgical findings r-RHA: replaced right hepatic artery; SMA: superior mesenteric artery; r-LHA: replaced left hepatic artery; CHA: common hepatic artery; ac-RHA: accessory right hepatic artery; MDCT: multi-detector computed tomographic

Celiac Artery Anatomy	MDCT angiography prevalence (percentage)	Surgical prevalence (percentage)	Correlation	Diagnosis	Surgery	Remark
Normal Trifurcation	33 (82.5%)	33 (82.5%)	100%	-	-	-
r-RHA from SMA	4 (10%)	4 (10%)	100%	Carcinoma Gall Bladder-3 Carcinoma Head of Pancreas-1	Extended Cholecystectomy Pancreatico-Duodenectomy	1.r-RHA Preserved 2.No Vascular Injury 3.No Oncological Compromise
r-LHA from Celiac Artery	1 (2.5%)	1 (2.5%)	100%	Carcinoma Head of Pancreas-1	Pancreatico-Duodenectomy	1.r-LHA Preserved 2.No Vascular Injury 3.No Oncological compromise
CHA from Aorta	1 (2.5%)	1 (2.5%)	100%	Carcinoma Head of Pancreas-1	Pancreatico-Duodenectomy	1.CHA Preserved 2.No Vascular Injury 3.No Oncological Compromise
ac-RHA from Proper Hepatic Artery	1 (2.5%)	1 (2.5%)	100%	Neuroendocrine Tumor of Pancreas with Left Lobe Liver Metastasis	Left Hepato-Pancreaticoduodenectomy	1.ac-RHA Preserved 2.No Vascular Injury 3.No Oncological Compromise

The statistical correlation showed that there was a 100% correlation between the preoperative MDCT findings and intraoperative surgical findings of the normal anatomical pattern and variations of the celiac artery and its branches. There was no statistical association of MDCT angiography findings as compared with the surgical findings using Pearson’s chi-square test and Fischer’s exact t-test. McNemar's test for significance was proved to have no significant changes in the MDCT angiography image and surgical findings. The agreement between CT image findings and intraoperative surgical findings was confirmed by Kappa statistics where it showed a Kappa value of 1.0, which indicates perfect 100% agreement.

## Discussion

The celiac artery is the first ventral branch of the abdominal aorta trifurcating into the left gastric artery (LGA), common hepatic artery (CHA), and splenic artery (SA), supplying the derivatives of foregut [15]. This classical branching pattern was first described as ‘tripus halleri’ by Albert Haller a Swiss anatomist in 1756 [16]. Since then, numerous variations in this branching pattern were reported, explained embryologically by the disappearance or retention of specific ventral segmental roots of the primitive arterial plexus by Tandler [1] in 1904. It was first classified into four types by Benjamin Lipshutz [5] in 1917 by 83 cadaveric dissections. In 1951, Michels [2] classified the celiac artery branches by 200 cadaveric dissections into six different types and aberrant hepatic arterial anatomy into 10 types, which is even now the most commonly followed classification system.

In the modern era of new technological advancements in radiology, the most novel and accurate approach to detect these variations are by non-invasive methods such as MDCT angiography [6-8]. It is now considered the gold standard in vessel imaging (accuracy 97%-98%) with significant advantages of simultaneous assessment of tumor size, the extent of involvement, lymph node status, and vascular encasement of the tumor [4,7,11]. Furthermore, intraoperative visualization of the surgical field is limited in patients with large tumors, peri-portal inflammation post biliary stenting, obesity, and dense adhesions due to previous surgery. In such cases, MDCT aids in preoperative visualization of the course of variant anatomy and planning of surgical procedures [4,9]. However few studies report that there is a chance of observer bias in detecting variations in MDCT, as it rarely presents cone-beam artifacts that are theoretically harmful causing image degradation [11]. But this has not been proven yet.

The absence of surgical confirmation of radiological findings is a major drawback in these MDCT studies [9]. In a study by Perwaiz et al. [10], in patients undergoing pancreaticoduodenectomy (PD), 28.3% of the patients were detected with variant anatomy during surgical dissections, which were missed during routine preoperative MDCT. Sahani et al. [11], in their study with 42 patients, compared the efficacy of MDCT in detecting variations of hepatic artery preoperatively and confirmed all his angiographic findings to correlate surgically. Surgical identification is now considered the gold standard, as a preoperative acknowledgment of these variations helps in planning an alternative strategy to approach the tumor without complications [4,10-13]. In our study, the radiological gold standard, preoperative MDCT, and the surgical gold standard, intraoperative correlation, were simultaneously used to identify the variations in celiac artery and hepatic artery branches and there was 100% correlation.

**Figure 6:**
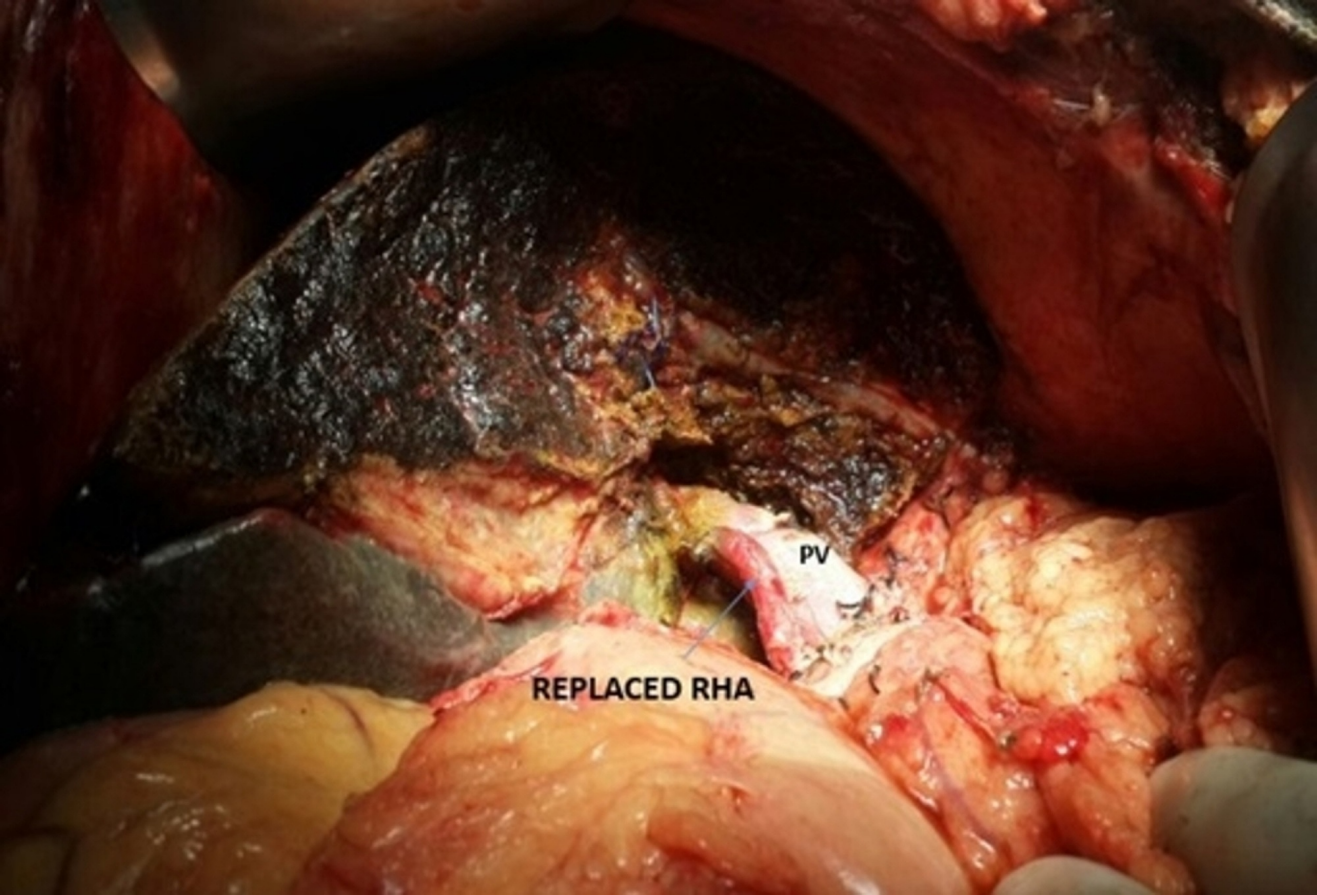
Intraoperative photograph showing the r-RHA coursing posterior to the main PV in the portocaval space r-RHA: replaced right hepatic artery; PV: portal vein

Celiac and hepatic artery variations found radiologically and intraoperatively correlate the findings (10%-45%) from other studies in the literature [2-4,6-12]. The most common variation was r-RHA arising from SMA (Figure 2), which is classified as Type 3 by Michel (11%) and Hiatt (10.6%) [2-3]. It has been found in many other studies at a frequency of 8%-20%) (Table 3) [2-4,8,11-13,17]. Embryologically, the persistence of the ventral longitudinal arterial segment connected to the SMA gives rise to the r-RHA [1]. The r-RHA was detected in three patients with carcinoma gall bladder and one patient with carcinoma head of the pancreas, who underwent extended cholecystectomy and PD respectively (Table 2). In all these patients, r-RHA from SMA was found coursing posteriorly to the pancreatic parenchyma and postero-lateral to the common bile duct in the hepatoduodenal ligament and finally supplying the right lobe of the liver. After complete Kocherization and lifting of the pars flaccida, the pulsations in the porta hepatis were felt and in none of these patients, tumor encasement over the r-RHA was found, making dissection possible with no oncological compromise (Figure 6). MDCT helped in providing a clear roadmap for dissection that obviates extensive dissection around the variant anatomy and averts vascular damage.

**Table 3:** Comparison with various other reported studies of variations of the celiac artery r-RHA: replaced right hepatic artery; SMA: superior mesenteric artery; r-LHA: replaced left hepatic artery; CHA: common hepatic artery; ac-RHA: accessory right hepatic artery; ND: not defined

Study	Year	Type	Country	Study Population	Normal Anatomy	Variations	r-RHA from SMA	r-LHA from Celiac Artery	CHA from Aorta	ac.RHA from Proper Hepatic Artery
Present Study	2016	Radiological + Surgical	INDIA	40	82.5%	17.5%	10.0%	2.5%	2.5%	2.5%
Michels et al [2]	1951	Cadaveric	USA	200	55.0%	45%	11.0%	ND	ND	ND
Hiatt et al [3]	1994	Surgical	USA	1000	75.7%	24.3%	10.6%	ND	0.2%	ND
Song et al [8]	2010	Radiological	South Korea	5002	89.1%	10.9%	ND	ND	0.4%	ND
Covey et al [17]	2002	Radiological	USA	600	61.3%	38.7%	12.2%	3.6%	2%	ND
Winston et al [4]	2007	Surgical	USA	371	51%	49%	15%	<1%	2%	ND
Abdullah et al [13]	2006	Surgical	France	932	68.1%	31.9%	10.2%	0.5%	0.3%	ND
Sahani et al [11]	2002	Radiological + Surgical	USA	42	59.6%	40.4%	11.9%	ND	ND	ND
Ram Mohan et al [12]	2014	Surgical	India	225	80.9%	19.1%	19.1%	ND	ND	ND

The r-RHA has a variable course around the pancreas such as the suprapancreatic, intrapancreatic, and, rarely, transpancreatic course, which may sometimes get encased by the tumor [8]. The degree of encasement of the vessel decides the resectability of the tumor [14]. Oncological compromise is the main concern in such cases. Expedient surgical plans, such as preservation or resection and anastomosis or graft placement, should be made [18]. Preservation of the variant arterial supply is imperative, as the dominant blood supply to the remnant bile duct and pancreatic stump post PD after gastroduodenal artery ligation is from the r-RHA. Any injury or complete resection of the variant vessel may lead to biliary and liver necrosis, especially in jaundiced patients with associated morbidity and mortality [19].

The replaced left hepatic artery (r-LHA) arising from the celiac artery was coursing to the right in the lesser sac through the fissure of ligamentum venosum and through the umbilical fissure supplying the left lobe of the liver. It was preserved as the patient underwent PD. The most common origin of r-LHA, which has been reported in the literature so far is from the LGA [2-4,8]. This rare variant of r-LHA arising from the celiac trunk was termed as ‘double hepatic artery’ by covey in 3.6% of patients [17]. It was also reported by Winston [4] and Abdullah [13] in 4% and 0.5% of patients in their study, respectively (Table 3). The prior idea of r-LHA is of great significance in the case of left hepatectomy, which facilitates minimal dissection around the porta-hepatis and avoids inadvertent ligation of these vessels causing left lobe necrosis [4].

**Figure 7:**
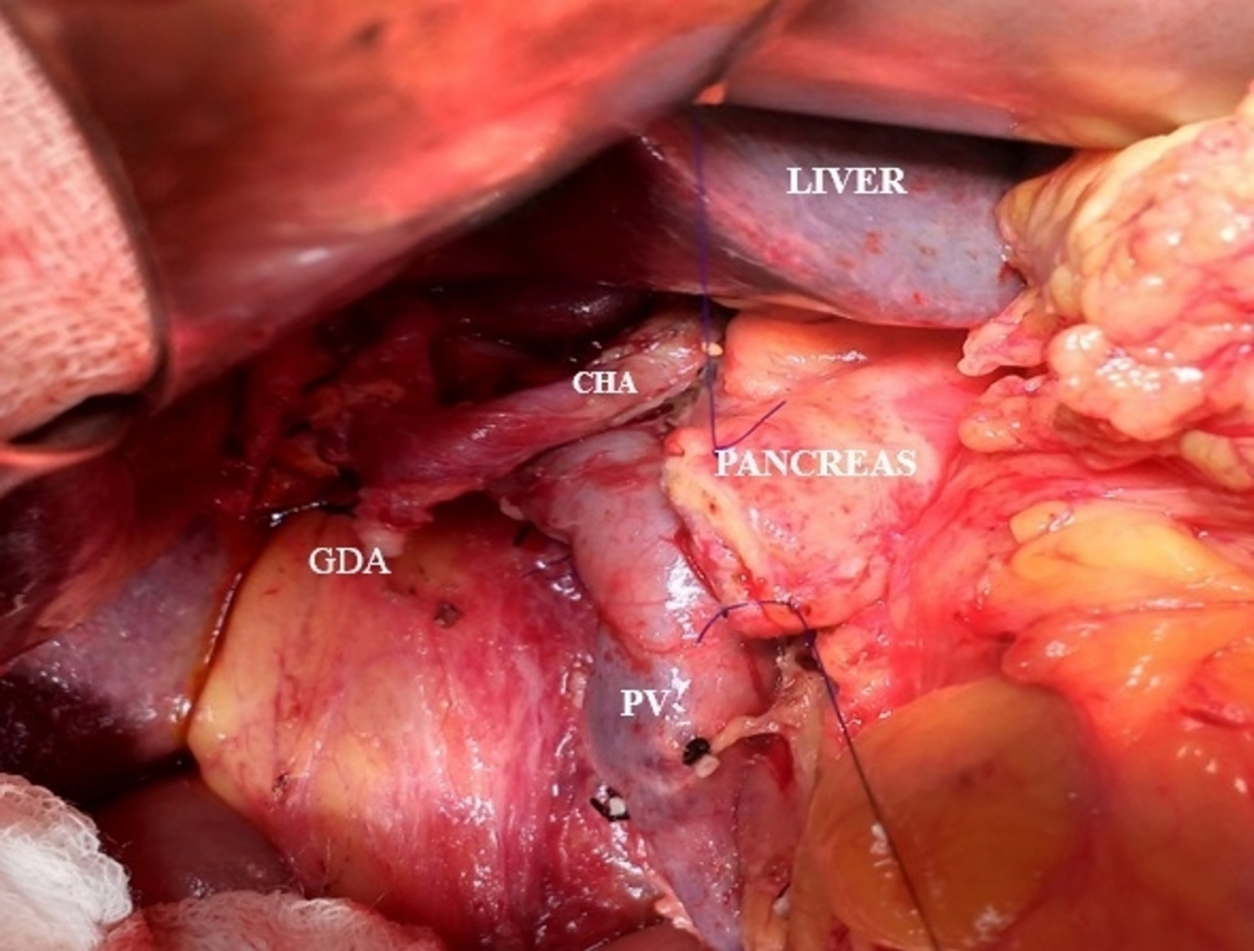
Intraoperative photograph showing the CHA arising from the abdominal aorta, crossing the PV anteriorly and ascending between the layers of the lesser omentum and in front of the epiploic foramen to enter the porta hepatis. The GDA appears ligated. CHA: common hepatic artery; PV: portal vein; GDA: gastroduodenal artery

In one patient who underwent PD for carcinoma head of the pancreas, the CHA was found to originate from the abdominal aorta and the LGA and SA arising as a common gastro-splenic trunk (Figure 7) was found crossing the portal vein anteriorly and ascending between the layers of the lesser omentum in front of the epiploic foramen to enter into the porta hepatis where it divides into the right and left hepatic arteries supplying the corresponding lobes of the liver. Its reported incidence in literature is around 0.2% to 2.5% and classified as Type 5 - Gastrosplenic Trunk in Michel’s study (Table 3) [2-4,8,13,17]. This variant was fortunately not involved by the tumor, hence was preserved with only ligation of the gastroduodenal artery (GDA) (Figure 7). However preoperative knowledge of its course enabled us for a confident dissection around the porta hepatis for safe lymphadenectomy.

Preoperative evaluation by MDCT abdomen identified an ac-RHA arising from the proper hepatic artery, which is an unrecognizable rare variant not yet described in the literature (Table 3), in a patient with a neuroendocrine tumor of the pancreas with left lobe liver metastasis (Figure 5). Hepaticopancreaticoduodenectomy was performed and was found coursing posterior to the main portal vein in the portocaval space and ascending posterolateral to the common bile duct, preserving this rare variant. The ac-RHA arises most commonly from SMA as reported by other studies [2-4,11]. Though theoretically it is believed that ac-RHA provides an additional blood supply to the right lobe, studies have revealed that it supplies specific territory intrahepatically, hence its compromise as an accessory artery in PD is questioned [20].

There were several limitations to this study. First, celiac artery stenosis, obstruction, and aneurysms were not identified in this study, which are significant. Second, postoperative complications were not studied, which quantifies the surgical impact of variant anatomy. Third, a larger sample size would have identified more variations.

## Conclusions

The variations of the celiac artery and hepatic arteries are not uncommon and their presence should not be undermined. The role and accuracy of MDCT angiography to assess the presence of variations of the celiac artery along with its relation to the tumor is evident from this study. Surgical confirmation of this preoperative radiological data has impressively helped in advance planning of surgical procedures to obviate inadvertent injury and disastrous postoperative complications and boost the moral confidence of the surgeon.
